# A Prospective Analysis of Scapular Positioning Patterns and Shoulder and Elbow Injury Susceptibility in Professional Baseball Pitchers

**DOI:** 10.3390/jcm14176267

**Published:** 2025-09-05

**Authors:** Kevin Laudner, Regan Wong, Keith Meister

**Affiliations:** 1 Hybl Sports Medicine and Performance Center, University of Colorado Colorado Springs, Colorado Springs, CO 80918, USA; 2 Texas Rangers Baseball Club, Arlington, TX 76011, USA; 3 Texas Metroplex Institute for Sports Medicine and Orthopedic Surgery, Arlington, TX 75033, USA

**Keywords:** baseball, dyskinesis, elbow, injury, scapula, shoulder, upper extremity

## Abstract

**Background/Objectives**: Baseball pitchers accumulate extreme upper extremity forces during the repetitive, high-velocity motion of throwing, which can lead to shoulder dysfunction and overuse injuries. Although scapular dyskinesis has been linked to various shoulder pathologies, there is a lack of evidence on the specific scapular patterns predisposing pitchers to injury. **Methods**: A total of 85 professional pitchers from a single professional baseball organization participated in the entirety of this study. All subjects had their scapular positions and motion patterns measured via a digital goniometer prior to the beginning of a competitive season. Scapular upward/downward rotation, anterior/posterior tilt, and internal/external rotation were assessed with the shoulder at rest and during elevation to 120° in the scapular plane. Overuse injuries of the shoulder/elbow sustained during the subsequent competitive season were documented by the team’s medical staff, with statistical comparisons between the injured (*n* = 34) and non-injured (*n* = 51) group for each scapular measure. **Results**: Pitchers who sustained shoulder/elbow injuries demonstrated significantly more scapular anterior tilt during humeral elevation compared to those without an injury (*p* = 0.04). The difference in anterior tilt between the two groups was 3.8° and had a medium effect size, suggesting clinical relevance. No significant between-group differences were found in any other scapular positions or motions (*p* > 0.22). **Conclusions**: Pitchers with increased scapular anterior tilt were more likely to sustain a shoulder/elbow injury, highlighting this kinematic feature as a potential risk factor. This finding suggests that anterior tilt might contribute to soft tissue strain, increasing injury susceptibility in pitchers.

## 1. Introduction

Shoulder and elbow injuries are a debilitating issue among baseball players, with pitchers experiencing a greater proportion of these injuries (67%) compared to position players (32%) [1]. These injuries often stem from the complex interaction of biomechanics, repetitive motion, muscular imbalances, and tissue tightness inherent to the sport [2]. Scapular dyskinesis, characterized by abnormal positioning or movement patterns, has long been associated with these injuries [3,4,5,6]. Although some research has prospectively investigated the effect of scapular dyskinesis as a whole in the development of injury among baseball players [7,8,9], there are currently no studies that have identified whether specific dyskinetic scapular patterns contribute to pathology. As such, understanding the connection between individual scapular motions and injury is crucial for refining effective prevention, evaluation, and rehabilitation strategies.

In baseball, the throwing side shoulder and elbow undergo extreme forces while throwing, particularly in pitchers who are also expected to repeat this ballistic activity hundreds of times during a game [10]. If kinematic disruptions occur in the complex sequence of events anywhere in the shoulder, then the repetitive and violent motion of pitching can lead to microtrauma and eventually overuse injuries if not managed properly. Research has highlighted the multifactorial nature of scapular dyskinesis in overall shoulder motion and adaptations and alterations that can occur in overhead athletes, including baseball pitchers, such as shoulder weakness [11,12], soft tissue tightness, and neuromuscular control deficits [13,14], leading to altered scapulothoracic rhythm [15]. These changes in scapular motion are believed to not only negatively affect performance [16] but may also increase a pitcher’s susceptibility to various shoulder and elbow injuries [5,17,18,19,20,21]. The consequences of such injuries can lead to excessive amounts of time lost from competition, surgery, and the inability of players to return to sport [22].

Despite advancements in biomechanical analysis, as well as prevention and rehabilitation protocols, there remains a critical gap in understanding whether aberrancies in specific scapular motions predispose baseball pitchers to shoulder and elbow injuries. Many studies have focused on general scapular biomechanics, clinical presentation, and clinical outcomes in individuals who already present with a pathologic condition. However, deeper exploration is necessary to elucidate these possible mechanisms of injury and guide targeted interventions and rehabilitation. Therefore, the specific aim of this study was to identify any scapular patterns that may contribute to the development of shoulder and elbow pathologies in professional baseball pitchers over the course of a competitive season.

## 2. Materials and Methods

Ninety-five minor league pitchers, taken from a sample of convenience from a single professional baseball organization, initially volunteered to participate in this study. At the time of testing, no subjects had sustained any injury within the last six months, and no subject had any history of spine or upper or lower extremity surgery as confirmed by the medical staff. Additional exclusion criteria consisted of any pitcher who threw less than 300 game-related pitches during the season, sustained an acute upper extremity injury or any spine or lower extremity injuries during the recorded competitive season, and players who were traded or released by the team. All subjects provided informed consent prior to any testing.

Initial measurements of scapular positioning were collected on day one of spring training. Following data collection, the team medical staff monitored each subject and documented any overuse injuries sustained during that same competitive baseball season (first day of spring training through the end of regular season competition, including playoff and championship games when appropriate). Thirty-four of these pitchers developed a single chronic shoulder or elbow injury during the competitive season that resulted in time being lost from competition, while 61 did not sustain an injury. Among the 61 subjects who did not sustain a shoulder/elbow injury, five pitchers threw less than 300 game-related pitches, and five sustained a lumbar or lower extremity injury, resulting in exclusion from the study. This resulted in 51 subjects in the non-injured group and a total of 85 subjects (Table 1). The levels of play for both groups can be viewed in Table 2, while the types of injury sustained by the experimental group can be viewed in Table 3. The five domestic levels of Minor League Baseball are, from lowest to highest levels of competition, as follows: Rookie League, Single-A, High-A, AA (or Double-A) and AAA (or Triple-A). Those players who sustained an injury during spring training had not yet been assigned to a level of play. Minor League Baseball also includes a foreign league (Dominican Summer League). The average number of days missed among the injured group was 55 days.

The specific aim of this prospective cohort study was to determine if baseball pitchers who sustained a pitching side shoulder or elbow injury during a competitive season presented with specific dyskinetic scapular patterns at the onset of spring training compared to those who remained injury-free. 3D scapular kinematics of the throwing arm were collected on the first day of spring training during the 2023 and 2024 seasons. Prior to data collection, all subjects provided informed consent in accordance with the university’s institutional review board guidelines. The same investigator conducted all scapular measurements.

Scapular kinematics of the throwing arm were measured using the EasyAngle^®^ Digital Goniometer (Meloq, AB, Stockholm, Sweden). This electronic goniometer is a small plastic device installed with a digital level and inertial measurement capabilities, allowing for measurement of all three orthogonal scapular planes.

**Figure 1:**
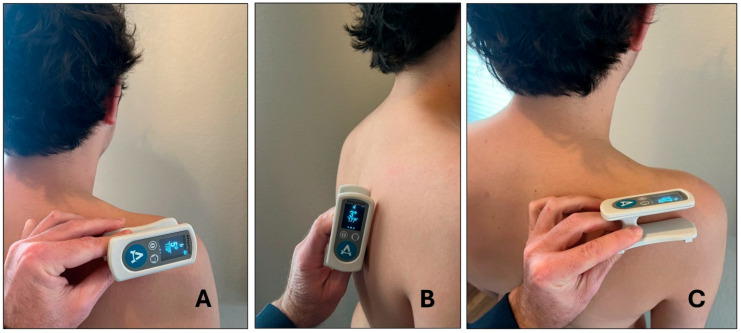
Placement of digital goniometer for scapular measurements ((**A**) upward/downward rotation; (**B**) anterior/posterior tilt; (**C**) internal/external rotation).

**Figure 2:**
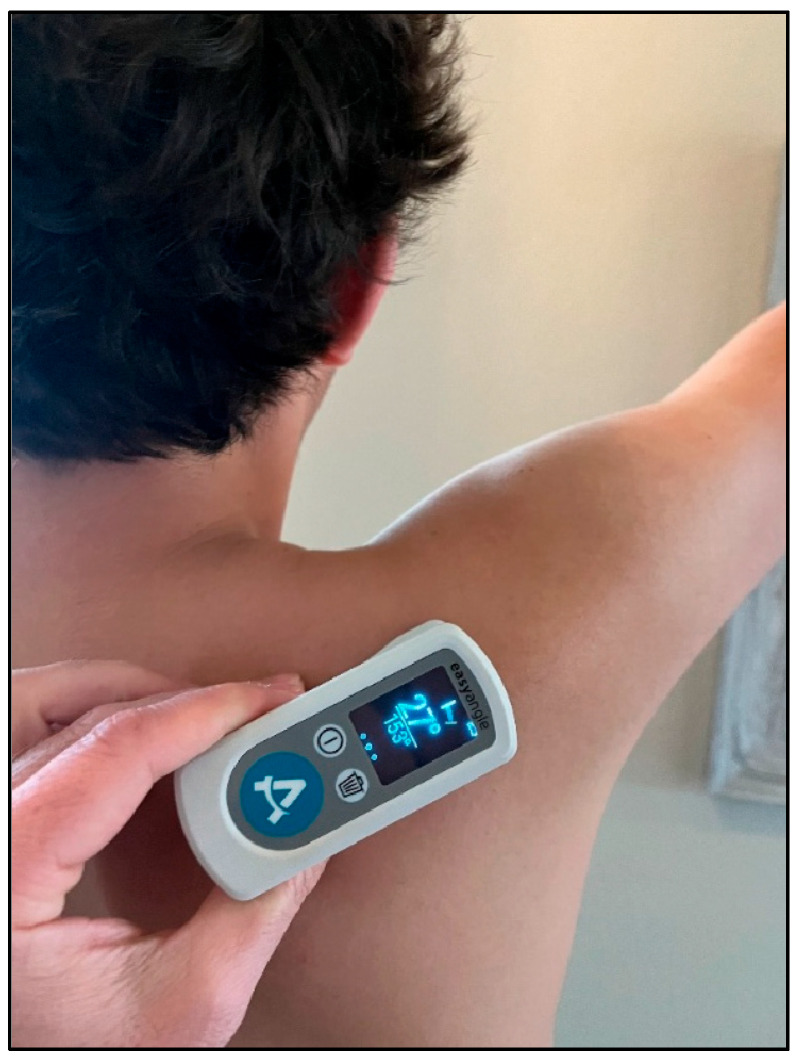
Scapular measurement of upward/downward rotation in elevated arm position.

For scapular testing, subjects stood in a relaxed position, and initially, with their throwing arm resting at their side. In this position, the face of the digital goniometer was placed with a posterior orientation along the spine of the scapula (Figure 1A) to measure the amount of scapular upward/downward rotation while at rest. Following this measurement, each subject was asked to actively elevate their arm up to 120° (measured with a goniometer) in the scapular plane (30° anterior to frontal plane), where scapular upward rotation was measured again (Figure 2). These steps were then repeated, altering the placement of the goniometer on the scapula with anterior/posterior tilt measured with the goniometer face oriented laterally on the medial scapular border (Figure 1B) and with internal/external rotation measured with the goniometer face oriented superiorly on the scapular spine (Figure 1C). The order of the measurements was randomized, and ample rest was provided between measurements to minimize fatigue.

All measurements were performed by the same investigator. We assessed the intratester reliability of this investigator performing these scapular assessments prior to testing using 10 subjects who were not part of the original study and who had no previous history of upper extremity injuries or surgeries. Each individual’s scapular motion was measured and then measured again within a 24 h period. The results demonstrated excellent intra-rater reliability and standard error of measurement for all three planes of motion (Table 4). This clinical scapular assessment has also been previously validated when compared to a 14-camera, 3-dimensional optical motion-capture system (ICC [2,3] range = 0.63–0.87) [23].

Frontal plane scapular motion was defined as scapular upward/downward rotation, sagittal plane movement was defined as anterior/posterior tilt, and motion in the transverse plane was defined as internal/external rotation. For each scapular plane, the scapula’s position at rest and the total arc of motion up to 120° of humeral elevation were calculated and used for statistical analyses. For the arc of motion calculation, the scapular position with the arm at rest was subtracted from the scapular position at 120° of elevation.

The dependent variables included resting scapular position for upward/downward rotation, anterior/posterior tilt, and internal/external rotation, as well as the total arc of motion for these three planes of motion. Separate two-tailed *t*-tests were performed to identify significant differences between the control and injured groups, using SPSS Statistics Software (Version 26; IBM, Armonk, NY, USA). A significance level of *p* < 0.05 was applied to all findings. To assess clinical significance, Cohen’s d effect sizes for scapular kinematics were calculated as (injured group—non-injured group)/standard deviation of the non-injured group. Effect sizes were categorized as small (0.20), medium (0.50), and large (0.80).

## 3. Results

There were no significant differences in subject demographics between groups (*p* > 0.26) (Table 1). Between-group differences for scapular position at rest and for total arc of motion can be found in Table 5. There were no significant differences between the groups in the resting position for any of the scapular planes (*p* > 0.22). Similarly, there were no significant differences in the arc of motion for upward/downward rotation (*p* = 0.63) or internal/external rotation (*p* = 0.23). However, there was a significant between-group difference for anterior/posterior tilt during the arc of motion, with the injured group demonstrating more anterior tilt than the control group (*p* = 0.04). The difference in anterior tilt between the two groups was 3.8°, which is larger than the 2.0° standard error of measurement demonstrated by the investigator of this study, who conducted all scapular assessments. This statistically significant difference also had a medium effect size, suggesting clinical relevance.

## 4. Discussion

Shoulder injuries in professional baseball pitchers represent a complex challenge, which can be exacerbated by scapular dyskinesis. Past research has demonstrated the common occurrence of scapular dyskinesis among patients with various shoulder and elbow injuries, such as rotator cuff tears [20], subacromial impingement [17,24,25], internal (posterior) impingement [5], SLAP tears [18], anterior glenohumeral laxity [21,26], and ulnar collateral ligament tears [19]. However, the role of aberrant scapular motion in the development of these upper extremity injuries is controversial and poorly understood [27,28]. The aim of this study was to investigate whether aberrancies in specific scapular motions were a contributing factor to shoulder and elbow injuries developed by professional baseball pitchers over the course of a competitive season. Our findings revealed that pitchers with more anterior tilt during overhead movement were more susceptible to sustaining a shoulder or elbow injury compared to pitchers with less anterior tilt. Both groups exhibited posterior tilting of the scapula during overhead elevation motion. However, the injured group exhibited more anterior tilt compared to the non-injured group. Although the difference in anterior tilt between the two groups was just 3.8°, the range of tilt in both groups was between −5.7 and −9.5°, so a 3.8° difference could be viewed as meaningful. Furthermore, this difference had a medium effect size (0.57), suggesting clinical relevance.

The observed association between excessive scapular anterior tilt and the development of shoulder and elbow injury aligns with previous literature, suggesting that this altered scapular motion can compromise the mechanics of the shoulder joint, leading to soft tissue damage [17,24,25,29]. Greater scapular anterior tilt is believed to place additional stress on the anterior glenohumeral structures, predisposing athletes to various overuse injuries [21,26].

The cause of the increased anterior tilt found in the injured pitchers in the current study could have been the result of several adaptations caused by the throwing motion. Because the pectoralis minor inserts onto the scapular coracoid process [30], tightness may result in pulling the scapula forward into anterior tilt. Borstad and Ludewig [31] found that a group of subjects with shorter pectoralis minor muscles had more anteriorly tilted scapulas compared to a control group. Weakness of the lower trapezius muscle may also play a role in increasing anterior tilt, as this muscle inserts at the base of the scapular spine and, when functioning properly, stabilizes and depresses the scapula, pulling the scapula back into a more posteriorly tilted position [32]. Posterior shoulder tightness in baseball pitchers has been extensively documented [33,34,35,36]. This tightness can stem from contractures and scar tissue of various soft tissue structures, such as the posterior glenohumeral capsule, teres minor, posterior deltoid, and infraspinatus, which work together to eccentrically control shoulder horizontal adduction and internal rotation during the follow-through phase of pitching [35,36,37]. Because these structures attach to the scapula, clinicians have described the effect this tightness can create by adaptively pulling the scapula into more anterior tilt [38,39,40], which may be especially prevalent in baseball pitchers during the follow-through phase of the throwing. Laudner et al. [34] supported this theory, reporting a moderate correlation between posterior shoulder tightness and forward scapular posture among professional baseball players.

The absence of significant findings in this study for any scapular position while at rest and for upward/downward rotation and internal/external rotation during movement may suggest that these variations in scapular motion do not have a direct impact on injury risk among professional pitchers. It is possible that elite athletes possess compensatory strategies that mitigate the detrimental effects of such dyskinetic movements, or that these variations are not inherently pathological in this population. More specifically, professional baseball pitchers may possess higher levels of physical conditioning and adaptive movement patterns, making them less vulnerable to certain types of scapular dysfunction. Additionally, the competitive nature of the sport may lead to rapid adjustments in mechanics or treatment interventions, minimizing the impact of scapular dyskinesis on injury risk during the season.

The identification of excessive scapular anterior tilt during overhead movement as a potential risk factor for injury highlights an important area for targeted interventions in professional baseball pitchers. Clinicians should consider incorporating assessments of scapular positioning, particularly anterior tilt, into preseason screenings and ongoing evaluations throughout the season. Corrective exercises and stretches aimed at improving scapular control and mobility may help reduce injury risk in pitchers exhibiting this type of specific dyskinesis. The findings from the current study emphasize the need to further investigate how specific types of scapular dyskinesis, rather than scapular dyskinesis as a whole [41], might contribute to injury mechanisms in overhead athletes.

This study has some limitations that should be considered when interpreting the results. The sample size, though representative of professional pitchers, may limit the generalizability of the findings to other levels of play or to other overhead athletes. Additionally, a post hoc power analysis was conducted and indicated that the study may have been underpowered to detect moderate differences in scapular upward/downward rotation (0.26) and internal/external rotation (0.21), and thus, these non-significant findings should be interpreted with caution. This also impacted the use of a more robust statistical analysis like a multivariate logistic regression. Our study was not powered for a multivariate analysis involving multiple covariates. For example, the level of play was noted at the time of injury but does not indicate the level where most of the pitches were performed. Therefore, the univariate test used provides a foundational analysis that supports future research using multivariable modeling with larger sample sizes and more detailed exposure data. Although all subjects had regular pitching rotations with their respective team, inevitably, they all had different pitch counts over the course of their season, which could have influenced injury outcomes. The relatively short duration of the study, limited to a single competitive season, may also have influenced the results. Shoulder and elbow injuries in baseball are often the result of cumulative microtrauma over several seasons, and a longer follow-up period might be required to capture the full extent of injury risk associated with scapular dyskinesis. Although all subjects had no recent history of injury to the upper extremity or any history of surgery, this study also does not exclude the possibility that the observed changes in anterior tilt may have resulted from previous injury. Lastly, bilateral scapular measurements were not collected. Differences in bilateral scapular motion may be adaptive or inherent alterations that allow for increased workload and performance or an increased risk of injury. Future research should consider longitudinal designs to investigate the long-term impact of scapular positioning on injury development and whether differences in bilateral scapular position and movement may increase the risk of pathology.

## 5. Conclusions

This study addresses the critical need to deepen our understanding of dyskinetic scapular patterns and their implications for injury. While pitchers who developed an injury did not show any differences in resting scapular position or for upward/downward rotation and internal/external rotation, they did present with more anterior tilt than the control group. These findings should be considered in the development of targeted pre-participation screening tools, personalized injury prevention training programs, and evidence-based rehabilitation protocols aimed at optimizing player health and performance. Future studies should explore the role of specific scapular abnormalities in injury development and consider multifactorial approaches that include biomechanical, workload, and historical injury data to better understand the complex relationship between scapular function and injury in overhead athletes.

## Figures and Tables

**Table 1:** Subject demographics (mean ± standard deviation).

	Injured Group (*n* = 34)	Non-Injured Group (*n* = 51)	*p*-Value
Age (yrs)	23.0 ± 2.2	23.4 ± 2.6	0.39
Height (cm)	188.5 ± 4.0	188.4 ± 5.8	0.94
Weight (kg)	84.8 ± 7.6	87.3 ± 10.4	0.26

**Table 2:** Different levels of play among both participant groups.

	Injured Group (*n* = 34)	Non-Injured Group (*n* = 51)
Spring Training	10 *	N/A
Dominican Summer League	1	0
Instructional league	4	14
Low A	7	4
High A	6	8
AA	1	16
AAA	5	9

* These pitchers sustained an injury during spring training, prior to being assigned to a specific level of play.

**Table 3:** Chronic shoulder/elbow injuries sustained during competitive season (*n* = 34).

Type of Injury	Number of Pitchers	Average Number of Days Missed
Rotator cuff lesion	9	25.9
Biceps tendinitis	5	17.5
Subacromial impingement/bursitis	5	27.5
Ulnar collateral ligament sprain	12	94.4
Elbow epicondylitis	1	34.0
Forearm strain	2	30.0

**Table 4:** Intra-reliability measurement for scapular assessment.

Total Arc of Scapular Motion	ICC	SEM (°)
Upward/Downward Rotation	0.83	4.1
Anterior/Posterior Tilt	0.91	2.0
Internal/External Rotation	0.85	3.0

ICC = intraclass correlation coefficient; SEM = standard error of measurement.

**Table 5:** Between-group descriptive statistics for scapular motion (mean ± standard deviation in degrees).

Test	Injured	Non-Injured	*p*-Value	Effect Size
*Scapular Position at Rest*				
Up/Dwn Rotation	5.9 ± 4.6	8.0 ± 8.9	0.24	0.24
Ant/Post Tilt	19.4 ± 7.4	21.4 ± 7.4	0.22	0.27
Int/Ext Rotation	31.6 ± 10.7	31.9 ± 9.3	0.93	0.03
*Total Arc of Scapular Motion*				
Up/Dwn Rotation	18.2 ± 9.1	17.2 ± 9.4	0.63	0.11
Ant/Post Tilt *	−5.7 ± 10.7	−9.5 ± 6.7	0.04	0.57
Int/Ext Rotation	−2.7 ± 9.0	0.6 ± 6.7	0.23	0.49

Up/Dwn = Upward/Downward; Ant/Post = Anterior/Posterior (negative value indicates posterior tilt); Int/Ext = Internal/External (negative value indicates external rotation). * Indicates statistically significant difference between groups (*p* < 0.05).

## Data Availability

The data in this study are unavailable due to privacy concerns.

## Abbreviations

The following abbreviations are used in this manuscript:

ICC	Intraclass correlation coefficient
SEM	Standard error of measurement
Up/Dwn	Upward/Downward
Ant/Post	Anterior/Posterior
In/Ext	Internal/External
